# Speed regulation strategy and algorithm for the variable-belt-speed energy-saving control of a belt conveyor based on the material flow rate

**DOI:** 10.1371/journal.pone.0247279

**Published:** 2021-02-22

**Authors:** Jianhua Ji, Changyun Miao, Xianguo Li, Yi Liu

**Affiliations:** 1 School of Mechanical Engineering, Tiangong University, Tianjin, China; 2 Department of Information Engineering, Tianjin University Renai College, Tianjin, China; 3 Tianjin Photoelectric Detection Technology and System Key Laboratory, Tiangong University, Tianjin, China; 4 Center for Engineering Internship and Training, Tiangong University, Tianjin, China; Tianjin University, CHINA

## Abstract

As an important transportation, the belt conveyor has been widely used and researched. It is urgent to solve the problem of energy saving and consumption reduction of belt conveyor. Aiming at reducing high energy consumption in the rated-speed operation of a belt conveyor, the present paper establishes an energy-saving belt-speed model of a belt conveyor using a polynomial regression-fitting algorithm and a small number of sample observations, and proposes a speed regulation strategy and particle swarm optimization–proportional–integral–derivative algorithm for the variable-belt-speed energy-saving control of a belt conveyor based on the material flow rate. The control strategy and algorithm adjust the running speed of the belt conveyor accurately according to changes in the material flow rate, thus reducing damage of frequent speed regulation to the belt conveyor and saving energy. Simulation analysis of a practical case shows that energy-saving belt-speed model, speed regulation strategy, and algorithm effectively reduce the energy consumption of a belt conveyor, and they thus have high application value in coal, ports, power, mine, metallurgy, chemical, and other industries. Further work in this field can be focused on the prediction of material flow rate of belt conveyor, the controllable adjustment duration of algorithm and the reduction of overshoot.

## 1 Introduction

The belt conveyor is used for continuous transportation in modern production. It has become one of the three main industrial transportation modes along with automobiles and trains, and has been widely used in coal, ports, electricity, power, mining, metallurgy, chemical, and other industries. It has been reported that 41% of global electricity is provided by coal-fired power plants, and coal is one of the main sources of carbon dioxide emissions worldwide [1]. Therefore, it is urgent to solve the problem of high energy consumption of belt conveyor.

Ji Jianhua *et al*. [2] theoretically derived an energy-saving control strategy for a belt conveyor system using the material flow rate and the design parameters of the belt conveyor. However, the design parameters of the belt conveyor are difficult to obtain, and the theoretical derivation is too cumbersome for engineering application. An on-line method of identifying parameters for an energy-saving model [3] has been proposed and achieves good results, but requires a large number of iterations and is time consuming. Wang Xiaowei *et al*. [4] established an energy-saving belt-speed model of the belt conveyor using a radial-basis-function neural network based on the operation sample data of a belt conveyor, but the model error is large.

Daijie He *et al*. [5, 6] studied the potential risks of regulating the speed of a belt conveyor and proposed solutions to mitigate those risks. Fuzzy proportional–integral–derivative (PID) algorithm [7, 8] and fuzzy algorithm [9–11] have been applied to the energy-saving regulation of the belt speed of a belt conveyor and provided good results. However, the design of fuzzy rules and membership functions of fuzzy algorithm is based solely on experience. There is thus a lack of systemization and it is difficult to deal with complex systems effectively. A genetic algorithm [12, 13] and artificial immune algorithm [14] have been used in the energy-saving belt-speed control of a belt conveyor, but these two algorithms require original information of the problem, have a large cost of numerical calculation, and have a low computational efficiency.

The present paper, on the premise of comparing the fitting results of polynomial regression and a back-propagation (BP) neural network, establishes an energy-saving belt-speed model of a belt conveyor adopting a polynomial regression-fitting algorithm with a small number of sample observations. Additionally, the paper proposes a speed regulation strategy and particle swarm optimization (PSO)-PID algorithm of the variable belt-speed control of a belt conveyor based on the material flow rate. Compared with previous studies, the research results in this paper can maximize energy savings, and are more suitable for engineering application.

## 2 Energy-saving belt-speed model of a belt conveyor

An energy-saving belt-speed model that accurately describes the relationship among the material flow rate, running speed, and system power consumption provides a basis for the energy-saving belt-speed regulation of a belt conveyor system. For the determined belt conveyor, the material flow rate and running speed together determine the power consumption of the belt conveyor

p=f(v,Q), (1)

where *v* is the running speed and *Q* is the material flow rate.

In terms of ensuring that normal production is not disturbed, the material flow rate cannot be adjusted artificially, and the magnitude and change in magnitude of the material flow rate must be passively accepted. Therefore, the only variable that can be adjusted artificially is the running speed.

The adjustment of the running speed is also constrained by the material flow rate. There is stacking if the material flow rate is high and the running speed is low whereas energy is wasted if the material flow rate is low (e.g., zero load) and the running speed is high (e.g., the rated speed). It is necessary to adjust the running speed according to changes in the material flow rate (i.e., obtain the energy-saving belt-speed model of the belt conveyor), to avoid the above two situations and to ensure that the belt conveyor runs at the most energy-saving speed.

The present paper adopts curve fitting to obtain the energy-saving belt-speed model of a belt conveyor for the purpose of facilitating engineering application. Sample data are selected according to the principles of integrity and comparability.

(1) Principle of sample data integrity

Sample data are taken evenly throughout the process from no load to full load of a belt conveyor to ensure the fitting effect.

(2) Principle of sample data comparability

Sample data with fewer observations are sampled equally from sample data with more observations to ensure the comparability of fitting results.

### 2.1 Algorithm design

The present paper fits the energy-saving belt-speed model of the belt conveyor adopting one-dimensional polynomial regression analysis and a BP neural network. Generally speaking, the fitting effect improves as the number of sample observations increases.

(1) Regression analysis of univariate polynomials

The advantage of polynomial regression analysis is that the curve fitting the sample observation values can be approximated by increasing the order of independent variables until the fitting is satisfactory. For the energy-saving model of a belt conveyor system, the running speed can be regarded a function of the material flow rate. It is therefore appropriate to fit the energy-saving belt-speed model of the belt conveyor by adopting univariate polynomial regression analysis.

(2) BP neural network analysis

The training strategy was main factors causing error. As the most widely used neural network model in artificial neural network (ANN), BP neural network is more accurate in the prediction of nonlinear physical parameters since the ability of optimizing weights and biases after sample training. In order to get more accurate predicting results, this paper uses BP neural network to analyze and predict the energy-saving belt-speed model of belt conveyor.

**Fig 1:**
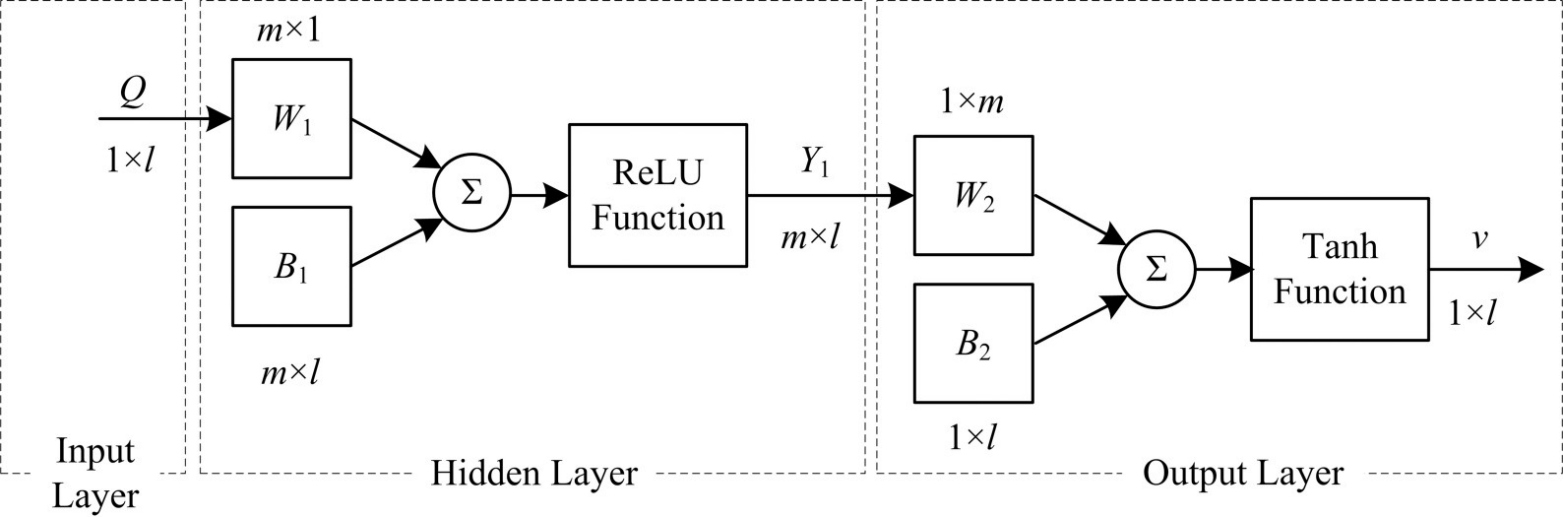
Structure of the BP neural network.

The BP neural network designed in this paper has three layers—an input layer, hidden layer, and output layer—with the hidden layer containing *m* neurons. The input and output of the BP neural network are the material flow rate *Q* and energy-saving running speed *v*, each expressed by a 1 ×*l* matrix (where *l* is the number of sample observations). The activation functions of the hidden layer and output layer of the BP neural network are the ReLU function and tanh function, where the weight matrices are the *m* × 1 matrix *W*_1_ and 1 × *m* matrix *W*_2_ and the deviation matrices are the *m*× *l* matrix *B*_1_ and 1 × *l* matrix *B*_2_. The structure of BP neural network is designed as shown in Fig 1.

Using the Python language and Tensorflow1.2 framework, the training set with 1400 sample data and the cross-validation set with 600 sample data are trained and verified according to the following steps.

① Reading of sample data
② Normalizing of input data
③ Design of the BP neural network
④ Design of the loss function
⑤ Implementation of a gradient descent algorithm
⑥ Iterative training to obtain the fitting value

When hidden neuron size changes, the average mean square error (*MSE*) and training time are shown in Table 1.

**Table 1:** *MSE* and training time of training set and cross-validation set at different hidden neuron size.

Hidden Neuron Size(×10^3^)	Training Set	Cross-validation Set	Training Time (s)
10	0.002617	0.003034	30.55
50	0.001592	0.001866	126.75
100	0.001303	0.001529	267.09
150	0.001364	0.001600	385.09
200	0.001396	0.001638	523.82
250	0.001391	0.001631	578.82
300	0.001336	0.001568	645.90
350	0.001210	0.001417	687.03
400	0.001198	0.001404	722.72
450	0.001215	0.001423	867.74
500	0.001207	0.001415	1225.05

Table 1 shows that with the increasing of the hidden neurons size, *MSE* of training set and cross-validation set is gradually reduced, the fitting effect is gradually improved, and the training time is increased. When the size of the hidden neurons is 400 thousand, *MSE* of training set and cross-validation set is the smallest, and the fitting effect is the best. The fitting effect decreases slightly when the size of the hidden neurons is more than 400 thousand. Considering the fitting effect and training time, the size of hidden neurons is determined to be 400 thousand.

### 2.2 Evaluation index

The root mean square error (*RMSE*) and determination coefficient (*R-squared*) are used to evaluate the fitting of the energy-saving belt-speed model [15–17].

A smaller value of *RMSE* indicates a better fitting and a stronger prediction ability of the model.

*RMSE* is calculated as

$$RMSE=\sqrt{\frac{\sum_{i=1}^{n}{\left(y_{i}-{y'}_{i}\right)}^{2}}{n}},$$ (2)

where *y*_*i*_ is the sample observation value, *y′*_*i*_ is the value predicted using the model, and *n* is the number of sample data.

The range of *R-squared* is [0, 1], with a value closer to 1 indicating better fitting.

*R-squared* is calculated as

$$R-squared=1-\frac{SS_{res}}{SS_{tot}},$$ (3)

where *SS*_*res*_ is the sum of residual squares while *SS*_*tot*_ is the sum of total deviation squares. This equation can be decomposed into

$$SS_{res}=\sum_{i=1}^{n}{\left(y_{i}-{y'}_{i}\right)}^{2},$$ (4)

$$SS_{tot}=\sum_{i=1}^{n}{\left(y_{i}-\bar{y}\right)}^{2},$$ (5)

where $\bar{y}$ is the mean value of the sample observations, calculated as

$$\bar{y}=\frac{1}{n}\sum_{i=1}^{n}y_{i}.$$ (6)

### 2.3 Performance analysis and model establishment

The rated running speed of a belt conveyor is *v*_*e*_ = 3.15 m/s, the belt width *B* = 1400 mm, the conveyor length *L* = 313.25 m, and the rated material flow rate is *Q*_*e*_ = 2000 t/h. The present paper uses five sets of sample data for prediction analysis, with the number of observations from the first set to the fifth set being 400, 200, 100, 50, and 25. Univariate polynomial regression fitting and BP neural network fitting are applied to the five sets of sample data. *RMSE* and *R-squared* are respectively given in Tables 2 and 3.

**Table 2:** *RMSE* of energy-saving belt-speed model fitting.

Fitting Method	Sample 1	Sample 2	Sample 3	Sample 4	Sample 5	Average
**Quartic Polynomial Fitting**	0.019187	0.019266	0.019455	0.019795	0.020876	0.019595
**Quintic Polynomial Fitting**	0.018581	0.018728	0.019015	0.019467	0.020709	0.019300
**Sixth Degree Polynomial Fitting**	0.015854	0.015896	0.016021	0.016177	0.016684	0.016126
**Seventh Degree Polynomial Fitting**	0.010713	0.010721	0.010774	0.010710	0.010911	0.010766
**Eighth Degree Polynomial Fitting**	0.008951	0.009012	0.009123	0.009173	0.009706	0.009193
**BP Neural Network Fitting**	0.035773	0.036104	0.036777	0.038067	0.041103	0.037565

**Table 3:** *R-squared* of energy-saving belt-speed model fitting.

Fitting Method	Sample 1	Sample 2	Sample 3	Sample 4	Sample 5	Average
**Quartic Polynomial Fitting**	0.999496	0.999457	0.999444	0.999421	0.999355	0.999435
**Quintic Polynomial Fitting**	0.999496	0.999487	0.999469	0.999440	0.999365	0.999451
**Sixth Degree Polynomial Fitting**	0.999633	0.999630	0.999623	0.999614	0.999588	0.999618
**Seventh Degree Polynomial Fitting**	0.999832	0.999832	0.999830	0.999831	0.999824	0.999830
**Eighth Degree Polynomial Fitting**	0.999883	0.999881	0.999878	0.999876	0.999860	0.999876
**BP Neural Network Fitting**	0.998131	0.998092	0.998013	0.997860	0.997498	0.997919

It can be seen from Tables 2 and 3 that the fitting effect of univariate polynomial and BP neural network becomes worse with the decrease of the number of sample observations: *RMSE* of fitting increases gradually and *R-squared* decreases gradually.

For BP neural network, due to the limited number of samples in the training set, the curve to be fitted is a combination of linear curves rather than complex nonlinear curves, and there is a discontinuity on the curve, so it fails to play its advantages of accurate prediction of nonlinear physical parameters. On the whole, the mean value of *RMSE* in BP neural network fitting for the five sets of sample data is 0.037565, while the mean value of *R-squared* is 0.997919. The fitting effect is worse than that of quartic polynomial fitting.

**Fig 2:**
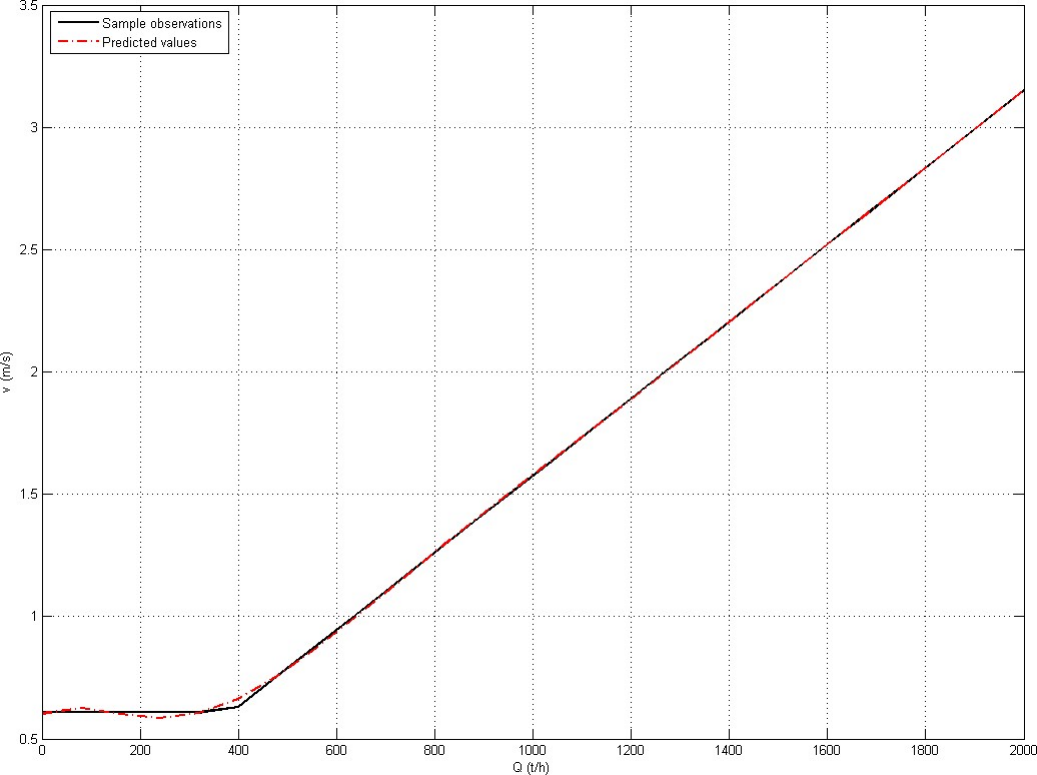
Eighth-degree polynomial fitting (25 observations).

For the univariate polynomial regression analysis method, with an increase in the highest order of polynomials, *RMSE* decreases, *R-squared* increases, and the fitting effect becomes better. The mean value of *RMSE* for the five sets of sample data reduces from 0.019595 for quartic polynomial fitting to 0.009193 for eighth-degree polynomial fitting, and the mean value of *R-squared* increases from 0.999435 for quartic polynomial fitting to 0.999876 for eighth-degree polynomial fitting. In eighth-degree polynomial fitting, *RMSE* is 0.009706 and *R-squared* is 0.999860 for sample 5 (25 observations), and the fitting effect is shown in Fig 2.

On the premise of ensuring the fitting accuracy, engineering application benefits from a shorter fitting time and fewer sample observations being required for the fitting of the energy-saving belt-speed model. In terms of the fitting effect and the number of required sample observations, the regression fitting of the univariate polynomial of sample 5 (25 observations) is suitable for engineering application.

The energy-saving belt-speed model is thus

$$v\left(Q\right)=\sum_{i=0}^{8}a_{i}Q^{i};\;0\leq Q\leq Q_{e},$$ (7)

where *a*_0_ = 0.6021409, *a*_1_ = 0.0010105, *a*_2_ = −1.2088216 × 10^−5^, *a*_3_ = 4.6496178 × 10^−8^, *a*_4_ = −7.6254949 × 10^−11^, *a*_5_ = 6.7652594 × 10^−14^, *a*_6_ = −3.3850711 × 10^−17^, *a*_7_ = 9.0010521 × 10^−21^, and *a*_8_ = −9.9055564 × 10^−25^.

It can be seen that the polynomial regression fitting algorithm proposed in this paper does not need complicated theoretical calculation, less sample observation data, better fitting effect, faster fitting speed and simpler and easier operation, which effectively reduces the complexity of obtaining energy-saving belt-speed model of belt conveyor.

## 3 Speed regulation strategy for a belt conveyor

The belt conveyor should adopt the following speed regulation strategy to avoid stacking and alleviate damage to the belt conveyor resulting from frequent speed regulation.

(1) Increases in the material flow rate

When the material flow rate increases, it is necessary to adjust the running speed of the belt conveyor according to the energy-saving belt-speed model immediately, and thus avoid a stacking accident due to the low running speed and the inability to transport material away in time.

(2) Decreases in the material flow rate

When the material flow rate is reduced and the energy-saving rate is more than 5% after speed regulation, the running speed of the belt conveyor is adjusted according to the energy-saving belt-speed model, to ensure the maximum energy-saving effect on the premise of reducing damage to the belt conveyor due to frequent speed regulation.

The energy-saving rate is

$$\Delta p\%=\frac{p_{1}-p_{2}}{p_{1}}\times 100\%,$$ (8)

where *p*_1_ and *p*_2_ are respectively the power consumption of the belt conveyor before and after speed regulation.

**Fig 3:**
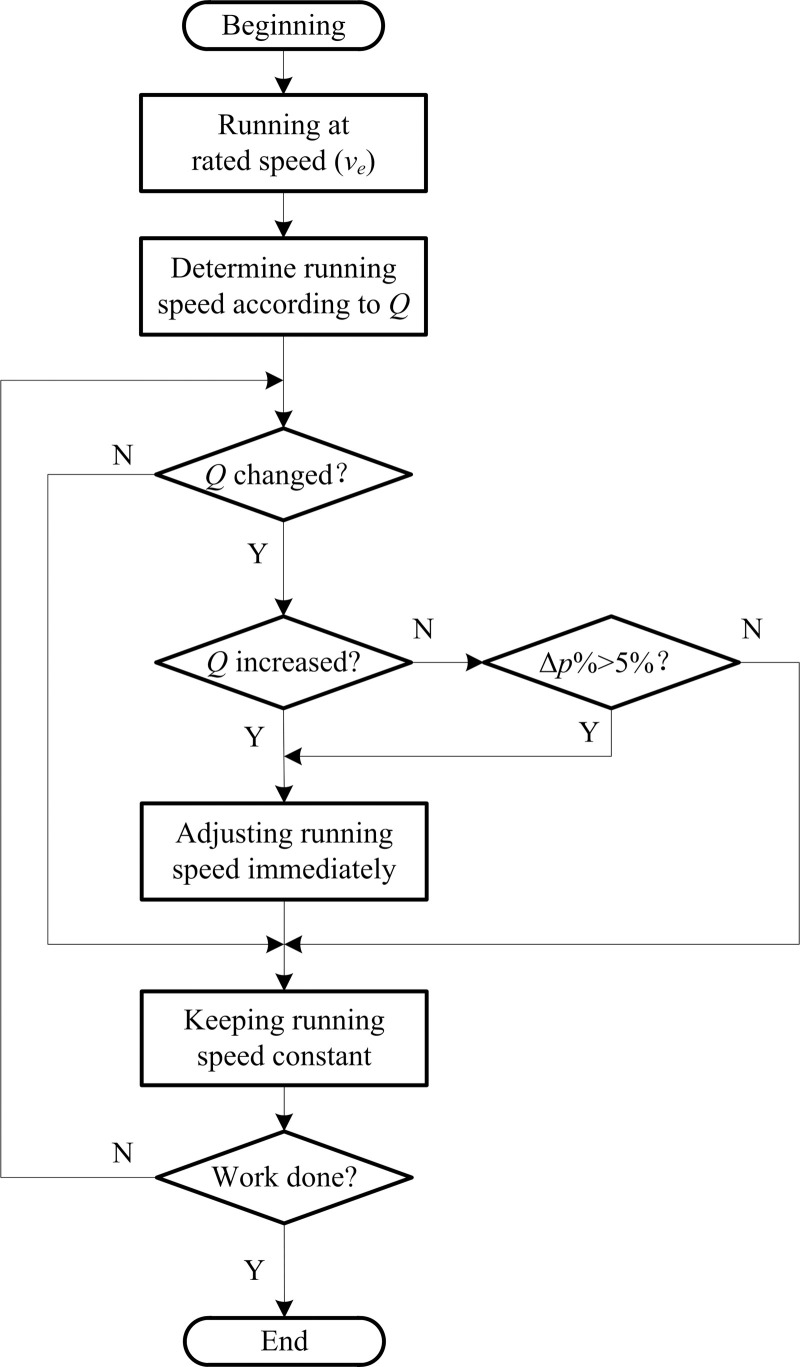
Flow chart of the speed regulation of a belt conveyor.

The flow chart of the speed regulation of a belt conveyor is shown in Fig 3.

## 4 Design of the PSO-PID algorithm

The PID algorithm is simple in principle, robust, and widely used, but the setting of the algorithm parameters is time consuming and laborious, the algorithm cannot guarantee the best control performance, and there is still much room for improvement in terms of the overshoot and adjustment duration.

The greatest advantage of the PSO algorithm is that it quickly obtains the global optimal solution of system parameters through information exchange among particles [18, 19]. The present paper adopts the PSO algorithm to optimize parameters of the PID controller; that is, the PSO-PID algorithm is used for speed regulation.

### 4.1 Algorithm design

The general form of the PID controller is

$$u\left(t\right)=K_{p}e\left(t\right)+K_{i}\int_{0}^{t}e\left(\tau \right)d\tau +K_{d}\frac{de\left(t\right)}{dt},$$ (9)

where *e*(*t*) is the system error while *K*_*p*_, *K*_*i*_, and *K*_*d*_ are respectively the weights of the system error, error integral, and error differential.

The value of the three parameters of PID controller is the decisive factor to ensure the control effect. When the proportional gain *K*_*p*_ increases, the response speed can be improved, but the stability is reduced and the oscillation is obvious. By integrating the integral function, the steady-state error can be effectively eliminated, but with the increase of the integral gain *K*_*i*_, the stability of the system and the response speed decrease. Increasing the differential gain *K*_*d*_ moderately can improve the stability of the system, but if *K*_*d*_ is too large, it will aggravate the oscillation of the system. Generally speaking, manual parameter tuning is time consuming and cannot guarantee the optimal control performance. At present, intelligent optimization algorithm is often used for parameter tuning.

The basic idea of PSO of PID controller parameters is as follows. First, the particle swarm is initialized to generate the velocity and position of all particles randomly. Particles in the particle swarm are then assigned to the parameters *K*_*p*_, *K*_*i*_, and *K*_*d*_ of the PID controller, and the SIMULINK model of the PID controller is run. Finally, the optimization is completed if the exit condition is satisfied. Otherwise, the speed and position of the particle are updated iteratively until the exit condition is satisfied, and the optimal solution is obtained.

The exit condition of PSO iterative optimization is that the value of the PID controller fitness function is less than or equal to a preset minimum fitness value or the preset maximum number of iterations is reached.

PSO updates the speed and location of particles according to

$$V_{t+1}=\omega V_{t}+c_{1}r_{1}\left(P_{t}-x_{t}\right)+c_{2}r_{2}\left(G_{t}-x_{t}\right),$$ (10)

$$x_{t+1}=x_{t}+V_{t+1},$$ (11)

where *ω* is the inertial factor, *c*_1_ and *c*_2_ are learning factors, *r*_1_ and *r*_2_ are random numbers in [0,1], *V* is the speed of particles, *x* is the position of particles, *P*_*t*_ denotes the current optimal position of particles, and *G*_*t*_ denotes the current best position of all particles in the particle swarm [20, 21].

The values of inertia factor *ω* and learning factors *c*_1_ and *c*_2_ directly affect the performance of PSO algorithm. The inertia factor *ω* reflects the inheritance of the particle to the current speed: when *ω* is larger, the global search ability of the particles is stronger; and when *ω* is smaller, the local search ability of the particles is stronger. The learning factors *c*_1_ and *c*_2_ respectively reflect the self-learning ability of particles and the ability of learning from the optimal particles: when *c*_1_ is larger, the self-awareness ability of particles is stronger, and it is easy to deviate from the optimal particles; when *c*_2_ is larger, the social cognitive ability of particles is stronger and it is easy to fall into local optimum. The best range of inertia factor ω is [0.4, 0.9], and the learning factors *c*_1_ and *c*_2_ are usually constant 2.

The integrated time and absolute error (ITAE) [22, 23] has good practicability and selectivity and is thus used as a fitness function to evaluate the optimization of PID parameters. It is defined as

$$J=\int_{0}^{\infty }t|e\left(t\right)|dt,$$ (12)

where *t* is the adjustment duration.

A smaller ITAE obviously corresponds to a better optimization effect.

**Fig 4:**
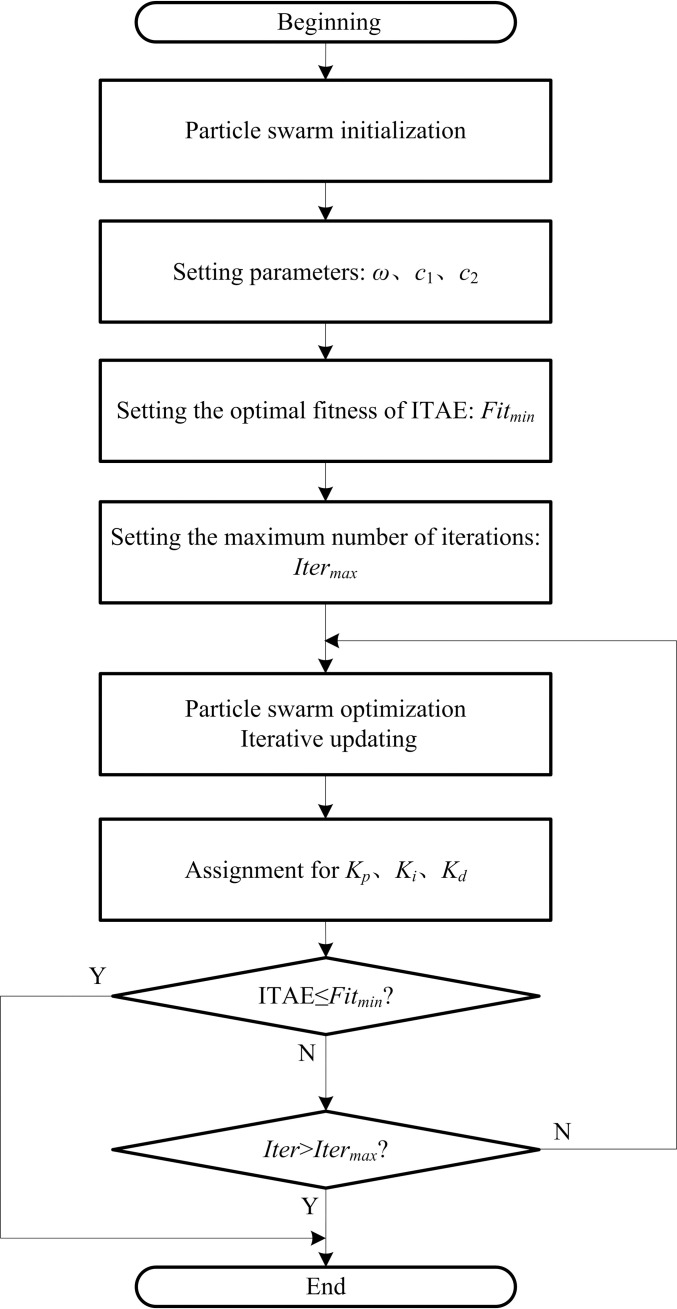
Flow chart of the optimization of PID control parameters.

The process of optimizing PID controller parameters is shown in Fig 4.

### 4.2 Performance analysis

The belt conveyor system can be approximately composed of inertia link and pure lag link, and its transfer function is approximately expressed as [4]

$$G\left(s\right)=\frac{e^{-2s}}{s\left(s+1\right)\left(s+5\right)}.$$ (13)

**Fig 5:**
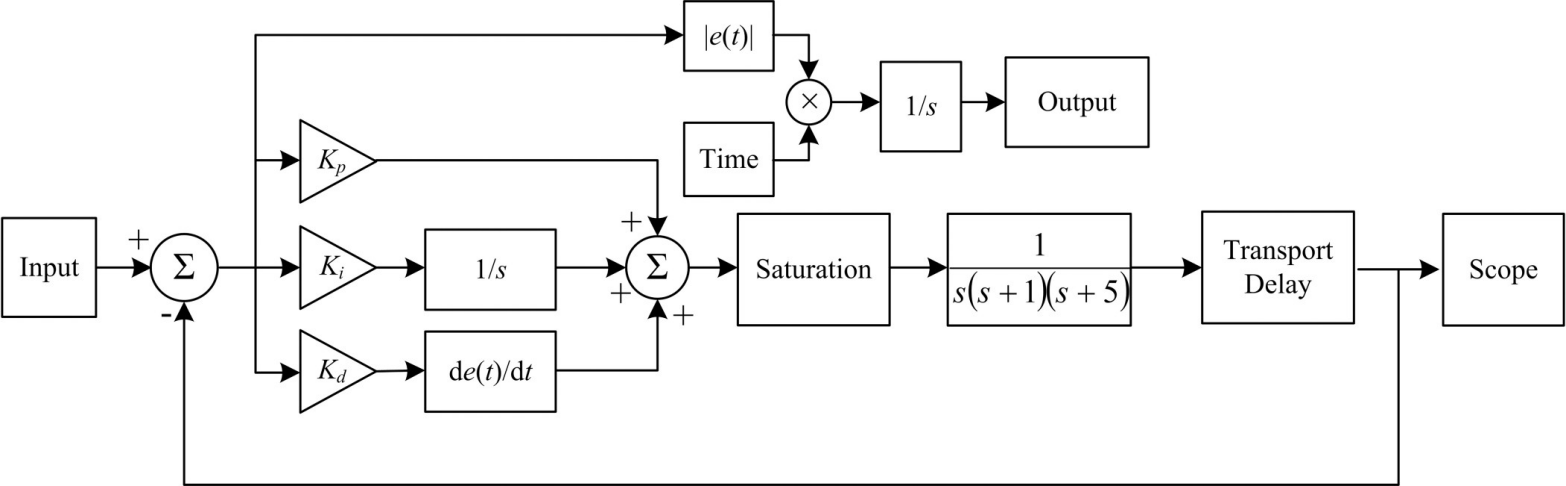
PSO-PID controller simulation schematic diagram.

Setting the main parameters of PSO algorithm to *ω* = 0.7, *c*_1_ = *c*_2_ = 2, swarmsize = 100, maxiter = 100, and minfit = 0.1. Where the swarmsize is the particle swarm size, the maxiter is the maximum number of iterations, and the minfit is the value of the fitness function. In the simulation environment of MATLAB 2014a, the PSO-PID controller simulation schematic diagram is shown in Fig 5.

**Fig 6:**
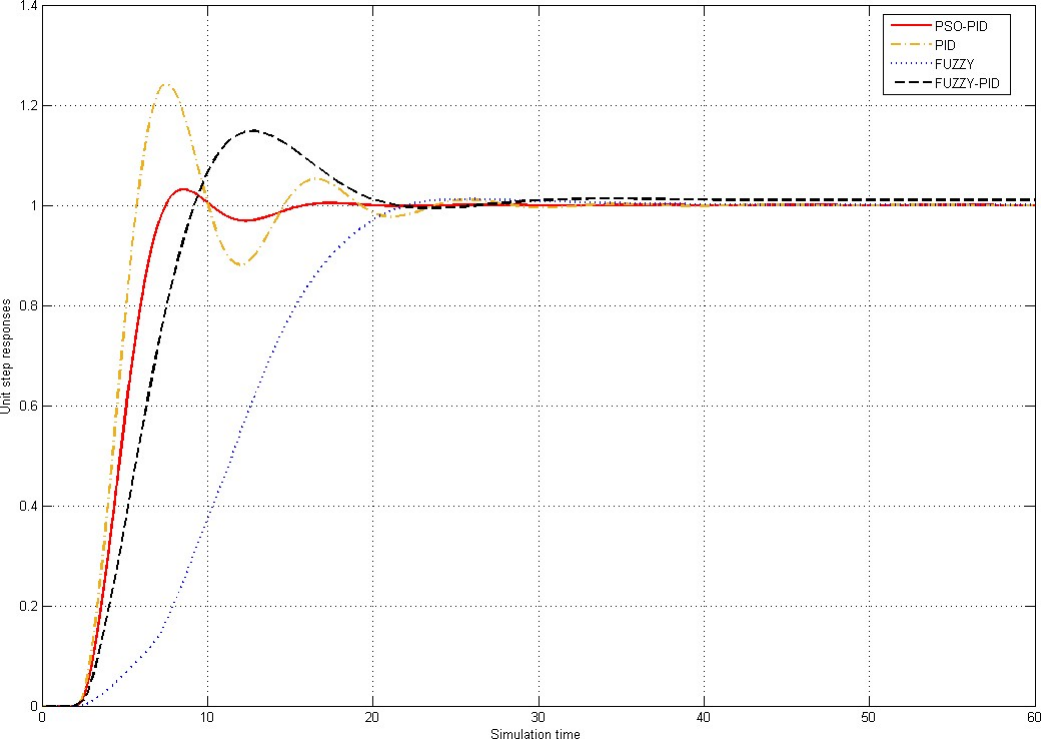
Curves of unit step responses of four algorithms.

The unit step response performance parameters of the fuzzy algorithm, the PID algorithm, the fuzzy PID algorithm, and the PSO-PID algorithm are given in Table 4 while the unit step response simulation curves of the four algorithms are shown in Fig 6. In the present paper, the time is the SIMULINK simulation time instead of the actual time, and the unit is thus not given.

**Table 4:** Performance parameters of unit step responses of four algorithms.

Algorithm	ITAE	Overshoot	Adjustment Duration	Steady-state Errors
**Fuzzy**	80.5362	1.20%	28.6704	0.00
**PID**	24.2279	24.24%	26.5814	0.00
**Fuzzy PID**	47.6669	14.81%	20.4619	0.01
**PSO-PID**	14.3325	3.15%	14.7620	0.00

The ITAE index, is related to the absolute error value in time, designing using the ITAE index may lead to small overshoots and damped oscillations. Therefore, it is strongly recommended to use it as an evaluation index for the comprehensive performance of the algorithm. When the ITAE is minimized, the transient response of the belt conveyor speed control system is improved in terms of maximum overshoot, settling time and rise time. It can be seen from Table 4 that the ITAE index of the four algorithms is in descending order: fuzzy algorithm (80.5362), fuzzy PID algorithm (47.6669), PID algorithm (24.2279), PSO-PID algorithm (14.3325). Therefore, the PSO-PID algorithm is of the best comprehensive performance.

The overshoot is the ratio of the instantaneous maximum deviation of the controlled variable to the steady value under the action of step input. It indicates the degree of controlled variable exceeding the set value, and the larger the overshoot, the more deviation the control result of the algorithm from the set value. The overshoot should be minimized to ensure that the belt conveyor will not cause serious consequences due to overspeed. It can be seen from Table 4 that the overshoot of the fuzzy algorithm and the PSO-PID algorithm have the same order of magnitude, and they are obviously better than the other two algorithms.

The adjustment duration refers to the shortest time for the controlled variable to return from the original stable state to the new equilibrium state after the control system is disturbed. The adjusting duration reflects the response speed and damping degree of the system. The shorter the adjustment duration is, the higher the work efficiency is. As shown in Table 4, the PSO-PID algorithm has the shortest adjustment duration, so the algorithm has the highest efficiency among the four algorithms.

The steady-state error is the deviation of the system when it transits from a steady state to a new steady state, or when the system is disturbed and rebalanced. The lower steady-state error of the control system is, the higher the control accuracy is. Therefore, the steady-state error is often used as an index to measure the performance of the control system. One of the tasks of control system design is to make the steady-state error as small as possible or less than a certain allowable limit value under the condition of considering other performance indexes. As can be seen from Table 4, only the steady-state error of fuzzy PID algorithm is non-zero, and that of the other three algorithms is zero among the four algorithms. That is, it reveals that the three algorithms perform well in maintaining the running speed of the belt conveyor.

The oscillation is a phenomenon that the size of controlled variable changes repeatedly and tends to be stable gradually. The larger the oscillation, the more frequently the controlled variable changes. For a belt conveyor, the more obvious the oscillation is, the more frequent the belt speed changes, and the more serious the damage of the motor to the belt conveyor. Therefore, the performance of the algorithm is gradually improved with the decrease of oscillation. It can be seen from Fig 6 that the amplitude and frequency of oscillation among the four algorithms is in descending order: the PID algorithm, fuzzy PID algorithm, PSO-PID algorithm, fuzzy algorithm.

**Fig 7:**
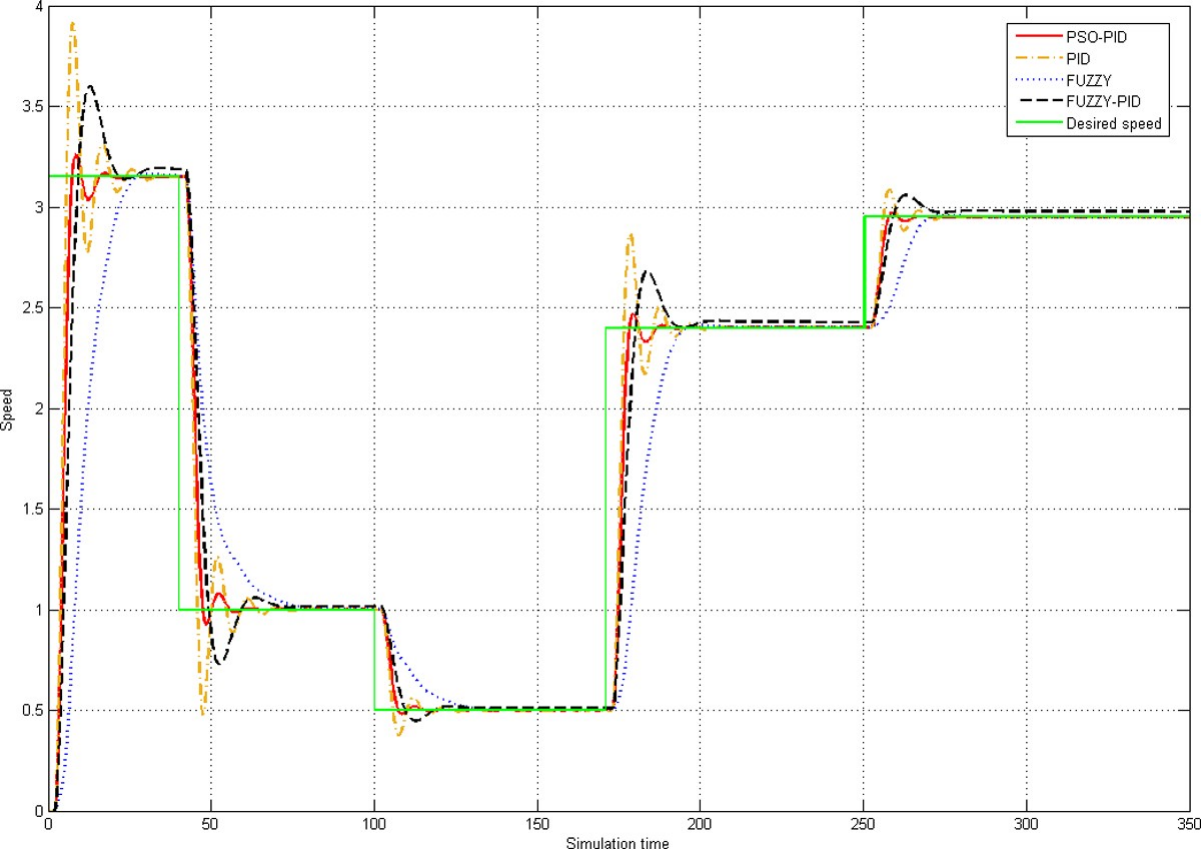
Tracking performances of four algorithms.

The tracking performance of the algorithm represents the ability of the control system to track the set point quickly, accurately, and stably. The tracking index is important to a belt conveyor having a variable belt speed. The tracking performances of the fuzzy algorithm, the PID algorithm, the fuzzy PID algorithm, and the PSO-PID algorithm are shown in Fig 7.

There are five speed adjustments in Fig 7: 0 to 3.15m/s, 3.15m/s to 1m/s, 1m/s to 0.5m/s, 0.5m/s to 2.4m/s, and 2.4m/s to 2.8m/s. The above speed adjustment takes full account of the difference in speed variation and shows the tracking performance of the algorithm in a more comprehensive way.

Fig 7 shows that the PSO-PID algorithm outperforms the other three algorithms in terms of the relative stability and adjustment duration. Compared with the other three algorithms, the PSO-PID algorithm can track the set point more quickly, accurately and stably, and has excellent tracking performance. It can also be seen from Fig 7 that although the overshoot of the fuzzy algorithm is zero, its adjustment duration is too long, so the tracking performance is poor.

To sum up, the PSO-PID algorithm is of the minimum ITAE and adjustment duration, the optimum tracking performances, zero steady-state error, the second smallest oscillation and overshoot in the start-up process. Therefore, the comprehensive performance of the PSO-PID algorithm is obviously better than the other three algorithms, and it is more suitable for belt conveyor speed control system.

Obviously, the PSO-PID algorithm proposed in this paper can automatically realize PID parameter tuning, greatly improves the control performance, and reduce the complexity of parameter tuning.

## 5 Analysis of simulation results in a practice case

The belt conveyor described in section 2.3 is taken as the object for analysis of the energy-saving effect. Table 5 gives energy-saving effects relative to the belt conveyor running at its rated speed.

**Table 5:** Analysis of the energy-saving effect.

Duration (*h*)	Material Flow Rate (*t*/*h*)	△*p*%	Belt speed (*m*/*s*)	Energy Consumption (*k*W·*h*)	Rated Energy Consumption (*k*W·*h*)	Energy Saving (*k*W·*h*)	Energy Saving Ratio (%)
0.5	1800	-	2.835	100	104	4	4.02
1	1750	1.46	2.835	196	204	8	4.07
0.5	1600	8.76	2.520	89	98	9	9.18
2	1200	22.75	1.890	276	345	69	19.88
0.5	1400	-14.79	2.205	79	92	13	13.83
1	1000	25.83	1.575	118	161	43	26.87
2	800	17.29	1.260	195	299	104	34.82
1	900	-10.40	1.418	107	155	48	30.73
3	600	27.43	0.945	234	413	179	43.38
0.5	200	35.37	0.608	25	57	32	55.99

In Table 5, △*p*% < 0 indicates that the material flow rate increases and the running speed needs to be raised immediately while △*p*% > 0 indicates that the material flow rate is reduced, but the running speed is reduced only when △ *p*% > 5%. The belt conveyor runs for 12 hours, the material flow rate changes nine times, and the running speed changes eight times. According to the speed regulation strategy, when the material flow rate reduces from 1800 to 1750 t/h, there is no speed regulation owing to △*p*% < 5%. During the operation of the belt conveyor, the total energy consumption is 1419 kW·h, the energy saving is 509 kW·h, and the average energy saving rate is thus 26.40%.

Mingming Huo *et al*. [9] set the material flow rate of the belt conveyor to 2000 t/h (rated material flow rate), 1500 t/h, 1000 t/h and 500 t/h, and run one hour at rated speed and energy-saving speed respectively, and the average energy saving rate of the energy-saving strategy is about 9.15%. According to the same experimental steps, the average energy saving rate of the proposed algorithm in this paper is about 18.09%. Obviously, the energy-saving effect of the proposed algorithm has been greatly improved.

## 6 Conclusion

This paper proposes a polynomial regression-fitting algorithm to establish the energy-saving belt-speed model of a belt conveyor, a speed regulation strategy and the PSO-PID algorithm for the energy-saving control of a belt conveyor having a variable belt speed based on the material flow rate. Analysis of an actual case reveals that the energy-saving belt-speed model has high accuracy, simple and easy to operate; the speed regulation strategy not only avoids the damage caused by frequent speed regulation to the belt conveyor, but also maximizes the energy saving effect; and the PSO-PID algorithm realizes automatic parameter tuning and achieves good results. The problem of energy saving and consumption reduction of belt conveyor discussed in this paper has become an important issue to be solved urgently. It is a major strategic demand of economic and social development, and has high engineering application value. Further work in this field can be focused on the prediction of material flow rate of belt conveyor, the controllable adjustment duration of algorithm and the reduction of overshoot.
